# Genetic deletion in uncoupling protein 3 augments ^18^F-fluorodeoxyglucose cardiac uptake in the ischemic heart

**DOI:** 10.1186/1471-2261-14-98

**Published:** 2014-08-08

**Authors:** Sara Gargiulo, Maria Piera Petretta, Adelaide Greco, Mariarosaria Panico, Michele Larobina, Matteo Gramanzini, Gabriele G Schiattarella, Giovanni Esposito, Mario Petretta, Arturo Brunetti, Alberto Cuocolo

**Affiliations:** 1 Department of Advanced Biomedical Sciences, University Federico II, Via Pansini 5, 80131 Naples, Italy; 2 CEINGE Scarl, Naples, Italy; 3 Institute of Biostructure and Bioimaging, National Council of Research, Naples, Italy; 4 Department of Translational Medical Sciences, University Federico II, Naples, Italy

**Keywords:** Uncoupling protein, Myocardial infarction, Glucose metabolism, Positron emission tomography

## Abstract

**Background:**

We investigated the effects of uncoupling protein 3 (UCP3) genetic deletion on ^18^F-fluorodeoxyglucose (FDG) cardiac uptake by positron emission tomography (PET)/computed tomography (CT) dedicated animal system after permanent coronary artery ligation.

**Methods:**

Cardiac ^18^F-FDG PET/CT was performed in UCP3 knockout (UCP3^−/−^) and wild-type (WT) mice one week after induction of myocardial infarction or sham procedure.

**Results:**

In sham-operated mice no difference in left ventricular (LV) volume was detectable between WT and UCP3^−/−^. After myocardial infarction, LV volume was higher in both WT and UCP3^−/−^ compared to sham animals, with a significant interaction (p < 0.05) between genotype and myocardial infarction. In sham-operated animals no difference in FDG standardized uptake value (SUV) was detectable between WT (1.8 ± 0.6) and UCP3^−/−^ (1.8 ± 0.6). After myocardial infarction SUV was significantly higher in remote areas than in infarcted territories in both UCP3−/− and WT mice (both p < 0.01). Moreover, in remote areas, SUV was significantly higher (p < 0.001) in UCP3−/− as compared to WT, while in the infarcted territory SUV was comparable (p = 0.29). A significant relationship (r = 0.68, p < 0.001) between LV volume and SUV was found.

**Conclusions:**

In a mice model of permanent coronary occlusion, UCP3 deficiency results in a metabolic shift that favored glycolytic metabolism and increased FDG uptake in remote areas.

## Background

The family of mitochondrial uncoupling proteins (UCP) has been recognized as being important in the regulation of mitochondrial function and reactive oxygen species (ROS) production [1]. In mitochondria from skeletal muscle, UCP3 was found to be necessary for the fasting-induced enhancement of fatty acid oxidation rate and capacity, possibly via mitigated mitochondrial oxidative stress [2]. Moderate physiological induction of UCP3 protein expression in muscle cells results in increased fatty acid oxidation in the absence of uncoupling, leading to the possibility that it may be involved in protection from lipotoxicity in muscle [3].

In the mammalian heart, UCP2 and UCP3 are the predominant isoforms [4] and have a protective effect in ischemia-reperfusion injury [5,6]. However, the role of UCP3 in cardiac muscle remains relatively unexplored and it is still controversial [7,8]. Animal models over- or under-expressing UCP3, evaluated by ex vivo genomic or proteomic analysis, did not provide uniform answers. Essop et al. [9] demonstrated a marked reduction in left ventricular (LV) UCP3 mitochondrial gene expression following experimental chronic hypoxia in association with metabolic switch from fatty acid to glucose utilization, resulting in an increased reliance on anaerobic glycolysis by cardiomyocytes. More recent evidences suggest that UCP3 genetic deletion promotes mitochondrial dysfunction, and increases ROS production and apoptotic cell death after myocardial infarction in mice, enlarging infarct size and accelerating heart failure [10]. However, the effects of UCP3 deletion on glucose metabolism after permanent coronary artery ligation have never been tested. In this study we measured in vivo ^18^F-fluorodeoxyglucose (FDG) cardiac uptake by high-resolution positron emission tomography (PET)/computed tomography (CT) in a mouse model lacking UCP3 after permanent coronary artery ligation to highlight possible alterations in myocardial energetic metabolism.

## Methods

### Animal studies

Animal experiments conformed to the “Guide for the Care and Use of Laboratory Animals” published by the US National Institutes of Health (NIH Publication No. 85–23, revised 1996) and were approved by the animal welfare regulation of University Federico II of Naples, Italy. Mice were purchased from the Jackson Laboratory (genetic background-strain: 129S4/SvJae). The UCP3 knockout (UCP3^−/−^) mice were obtained as previously described [11]. Male adult UCP3^−/−^ (aged 8 to 9 weeks, n = 17) and wild-type (WT) mice (aged 8 to 9 weeks, n = 14) were included in the study and maintained under identical conditions of temperature (21 ± 1°C), humidity (60 ± 5%), and light–dark cycle and had free access to normal mouse chow.

### Mouse model of myocardial infarction

Myocardial infarction was induced in UCP3^−/−^ (n = 8) and WT mice (n = 8) by permanent ligation of the left coronary artery. Sham-operated animals underwent the same procedure without ligation of the coronary artery at the same time (sham: UCP3^−/−^, n = 9 and WT, n = 6). Permanent ligation of left coronary artery was performed as previously described [12] using a surgical microscope to clearly detect and ligate the small vessel, dedicated microsurgical instruments, thin sutures and needles, and a customized mouse ventilator (Harvard Apparatus, March-Hugstetten, Germany). Briefly, mice were anesthetized with 2.4% sevofluorane plus oxygen, fixed in a supine position on a heating table to prevent hypothermia, intubated and ventilated with a tidal volume of 200 μl and a respiratory rate of about 110 breaths/min. The thoracotomy was performed by a transverse 5 mm incision of the left fifth intercostal space, 2 mm away from the left sternal border, then the pericardial sac was opened and the left anterior descending coronary artery was occluded 2–3 mm distal to the tip of the left auricle using a 7.0 silk suture.

### Trans-thoracic echocardiography

Trans-thoracic echocardiography was performed 7 days (range 5–8) after surgery in both myocardial infarction and sham groups using the Vevo 770 high-resolution imaging system (VisualSonics), as previously described [13]. Briefly, the mice were anesthetized with an intramuscular injection of ketamine 100 mg/kg and xylazine 2.5 mg/kg, and echocardiograms were performed with a 30-MHz RMV-707B scanning head. Cardiac function was evaluated by measuring LV fractional shortening [13].

### PET/CT imaging

The same day of echocardiography, PET/CT was performed in all mice using a dedicated animal scanner (eXplore Vista, GE Healthcare, Milwaukee, WI, USA). The scanner has a PET spatial resolution of 1.6 mm full-width at half maximum and a CT spatial resolution of 200 μm. The animals had unrestricted access to water and their normal food before scanning. Prior to imaging, mice were warmed for 15 minutes with an infrared lamp to induce vasodilatation of the lateral tail vein. Mice were then anesthetized with a mixture of isoflurane 4% and oxygen 1 L/minute for 5 minutes and positioned in the mouse restrainer. A dose of 300 MBq/kg of ^18^F-FDG was administrated as a bolus in the lateral tail vein by a 30-gauge needle (injection volume, 100 μL). Animals were maintained at a temperature of 23°C during the biodistribution of FDG. This standardized protocol safeguarded animal welfare and optimized the PET scan with FDG, i.e., avoiding stress for restrain or cold to reduce interscapular brown fat uptake and improve the uptake in the target structure. In addition, it minimizes the risk of motion artifacts during acquisition. After 40 minutes, mice were anesthetized with ketamine 100 mg/kg and xylazine 10 mg/kg (injection volume, 100 μL/10 g). Thereafter, the mice, with the heart centered in the tomograph, were symmetrically positioned on a warm bed with micropore tape, and a 15-minute static PET (single bed position with an axial field-of-view of 4.8 cm; energy window 250–700 keV) scan was performed, followed by a 7-minute CT scan.

PET/CT images were processed as previously described [14]. PET data were reconstructed using a 3D-FORE/2D-OSEM iterative algorithm (16 subsets, 2 iterations, matrix size 175 × 175, voxel size of 0.3875 × 0.3875 × 0.7750 mm^3^) including random, scatter correction, dead time, decay, and attenuation correction using CT data. Reconstructed images were reoriented to obtain axial sections perpendicular to the LV long axis and the whole ventricle wall was manually segmented tracing a region of interest in each slice (eXplore Vista Software). FDG uptake was measured in the LV wall volume and expressed as standardized uptake value (SUV): tissue activity (MBq/cc)/[injected dose (MBq)/body weight (g)]). Ellipsoidal regions of interest were also drawn on the right lobe of the liver and on the left triceps brachii muscle and FDG uptake was expressed as average SUV. In UCP3^−/−^ and WT mice with myocardial infarction, SUV was also measured separately in the infarcted territory and in remote areas. Automated image analysis software (MunichHeart) was used to measure LV volume and infarct size on the basis of volumetric sampling of tracer uptake [15,16]. This software allowed long-axis definition, volumetric polar map calculation, and report page generation for the database. Each polar map was normalized to its maximum uptake value. Extent of infarct was expressed in percentage value (% defect area/LV area) by counting the elements in the polar map with an activity below a threshold (50% of the maximum) and relating this value to the total number of polar map elements. The reproducibility and accuracy of this approach for measurement of infarct size in a mice model of permanent coronary occlusion have been documented [14].

### Statistical analysis

All data were expressed as mean ± standard deviation. Comparisons between two groups were performed using the unpaired Student *t* test. Two-way analysis of variance was performed to analyze differences by genotype and myocardial infarction among the four groups, including a Tukey post-hoc analysis if a significant F test occurred. Linear regression analysis was performed to evaluate the relationship between LV volume and SUV. A p value < 0.05 was considered statistically significant.

## Results

**Figure 1:**
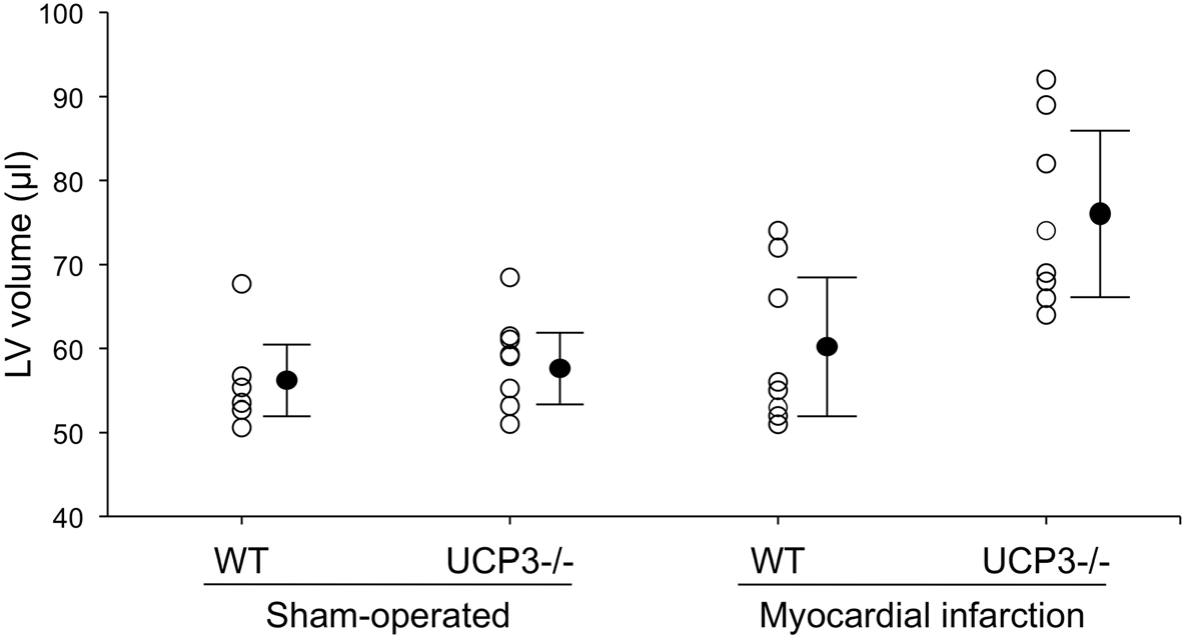
**Individual values for LV volume in sham-operated and myocardial infarction WT and UCP3**^**−/− **^**mice.** Closed circles indicate mean ± standard deviation.

Individual values of LV volume in the four groups of mice are illustrated in Figure 1. In sham-operated mice no difference was detectable between WT (56.1 ± 6.1 μl) and UCP3^−/−^ (58.7 ± 5.1 μl). After myocardial infarction, LV volume was higher in both WT (59.9 ± 9.3 μl) and UCP3^−/−^ (75.5 ± 10.8 μl) as compared to sham animals, with UCP3^−/−^ mice showing the highest values. At two-way analysis of variance a significant interaction (p < 0.05) between genotype and myocardial infarction was found (Table 1).

**Table 1:** Effects of genotype and myocardial infarction and their interaction on left ventricular volume at two-way analysis of variance

	**Sum of square**	**Degree of freedom**	**Mean square**	**F-value**	**p-value**
Model	1782	3	594	8.78	0.0003
Genotype	627	1	627	9.27	0.005
Myocardial infarction	805	1	805	11.9	0.002
Genotype/myocardial infarction	323	1	323	4.78	0.04
Residual	1826	27	68		
Total	3608	30	120		

**Figure 2:**
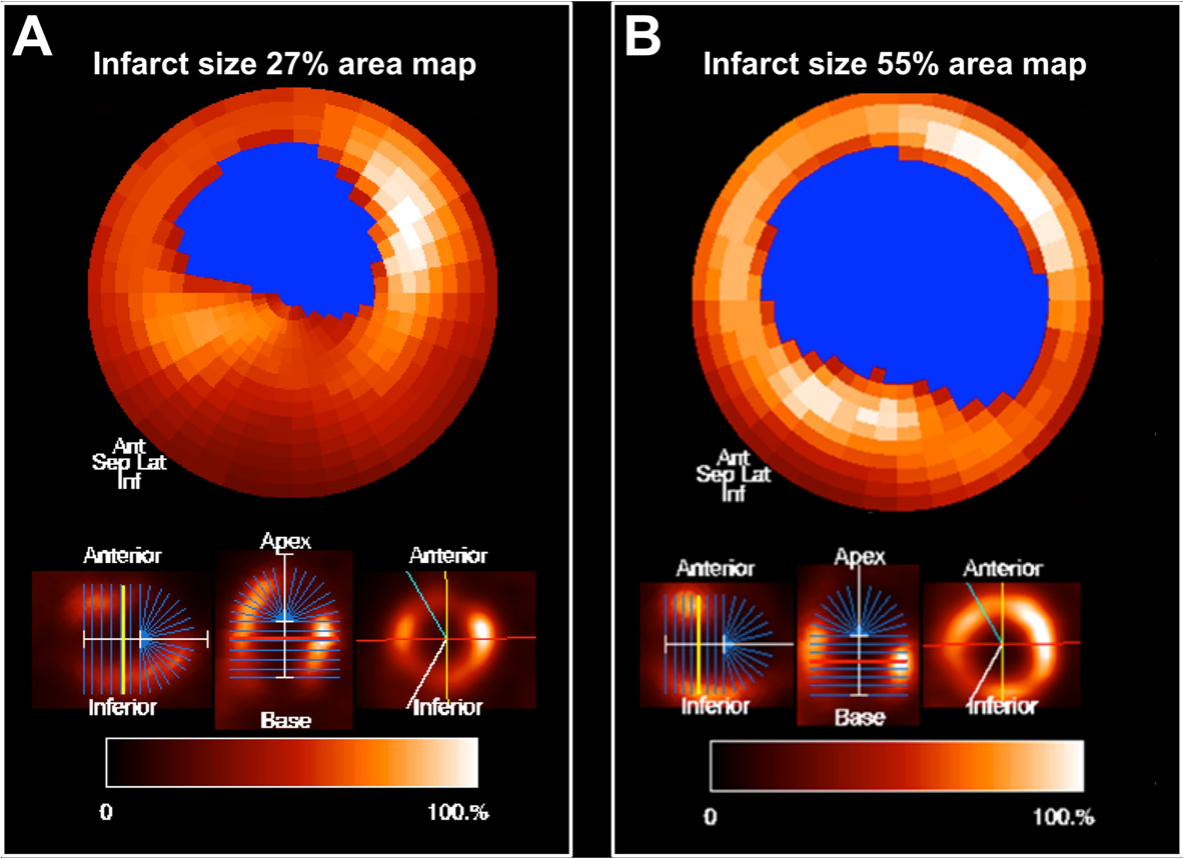
**Vertical long-axis, horizontal long-axis, and short-axis slices (on the bottom) and the resulting polar map (on the top) in a WT mouse (A) and an UCP3**^**−/− **^**mouse (B) after myocardial infarction.**

At trans-thoracic echocardiography, sham-operated UCP3^−/−^ mice showed LV fractional shortening comparable to WT (56.5 ± 4.6% and 54.1 ± 4.2%, respectively, p = NS). After myocardial infarction, LV fractional shortening was significantly lower (p < 0.05) in both WT (42.7 ± 3.1%) and UCP3^−/−^ (24.4 ± 2.5%) mice compared to sham-operated animals. UCP3^−/−^ mice displayed a significant worsening in cardiac function after coronary artery ligation compared with WT (p < 0.05). Furthermore, UCP3 genetic deletion increased infarct size after coronary artery ligation as compared to WT (44 ± 9% vs. 29 ± 7%, p < 0.005). Vertical long-axis, horizontal long-axis, and short-axis slices and the resulting polar map in a WT mouse and an UCP3^−/−^ mouse after myocardial infarction are illustrated in Figure 2.

**Figure 3:**
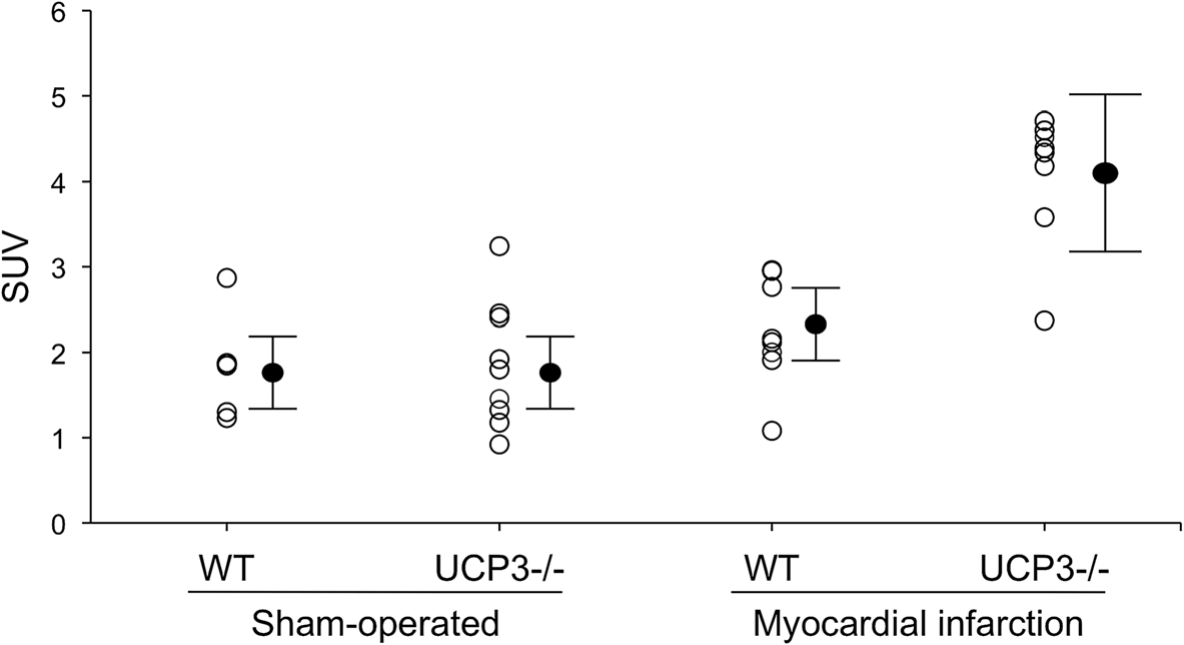
**Individual values for SUV in sham-operated and myocardial infarction WT and UCP3**^**−/− **^**mice.** Closed circles indicate mean ± standard deviation.

**Figure 4:**
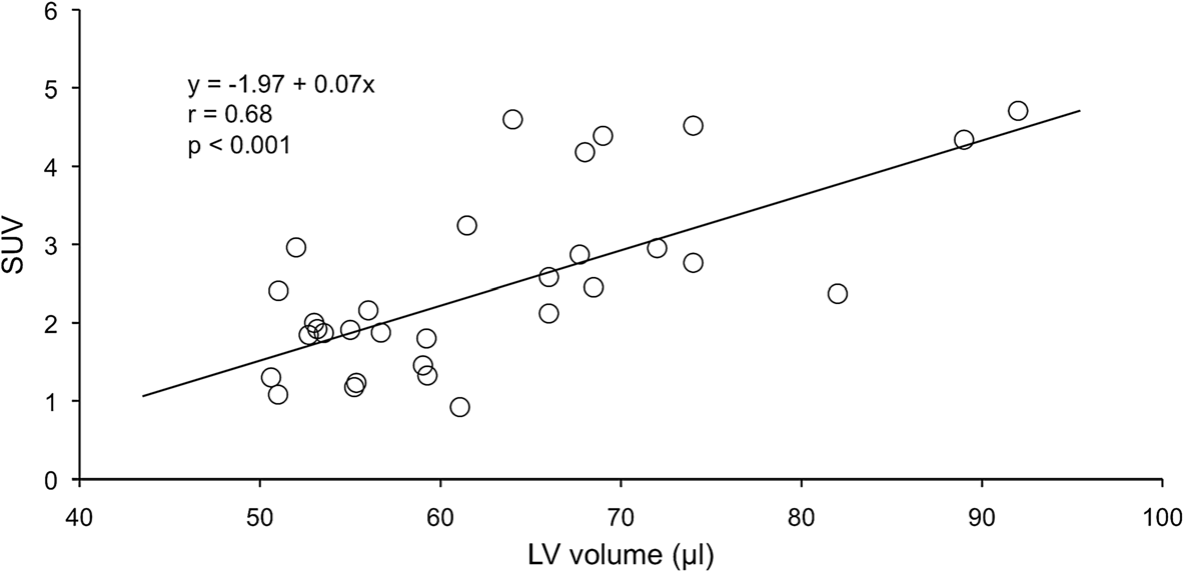
Relationship between LV volume and SUV at linear regression analysis.

Figure 3 shows individual values of SUV in the four groups of mice. In sham-operated animals no difference was detectable between WT (1.8 ± 0.6) and UCP3^−/−^ (1.8 ± 0.6). After myocardial infarction, SUV was higher in both WT (2.2 ± 0.6) and UCP3^−/−^ (4.0 ± 0.9) compared to sham animals, with UCP3^−/−^ mice showing the highest values. The results of two-way analysis of variance are reported in Table 2. As shown, a significant interaction (p < 0.005) between genotype and myocardial infarction was found. At linear regression analysis a significant relationship (r = 0.68, p < 0.001) between LV volume and SUV was found (Figure 4). At separate analysis, after myocardial infarction SUV was significantly higher in remote areas than in infarcted territories in both UCP3^−/−^ and WT mice (Table 3). In remote areas, SUV was significantly higher (p < 0.001) in UCP3^−/−^ as compared to WT, while in the infarcted territory SUV was comparable (p = 0.29) in the two groups of mice. Finally, in non-cardiac tissue (liver and muscles) SUV was independent from UCP deletion and myocardial infarction (Table 4).

**Table 2:** Effects of genotype and myocardial infarction and their interaction on SUV at two-way analysis of variance

	**Sum of square**	**Degree of freedom**	**Mean square**	**F-value**	**p-value**
Model	24	3	8.0	14.4	0.0001
Genotype	5.8	1	5.8	10.3	0.003
Myocardial infarction	12	1	12	21.6	0.0001
Genotype/myocardial infarction	5.4	1	5.4	9.79	0.004
Residual	15	27	0.56		
Total	39	30	1.30		

**Table 3:** **SUV values in infarcted territory and remote areas in WT and UCP3**^**−/− **^**mice after myocardial infarction**

	**Infarcted territory**	**Remote areas**	**p-value**
**WT**	1.3 ± 0.3	2.5 ± 0.7	<0.01
**UCP3**^**−/−**^	1.7 ± 0.9	4.3 ± 1.0*	<0.01

*p < 0.001 WT vs. UCP3^−/−^.

**Table 4:** **Liver and muscle SUV values for sham-operated and myocardial infarction WT and UCP3**^**−/− **^**mice**

	**Sham-operated**		**Myocardial infarction**			
	**WT**	**UCP3**^**−/−**^	**WT**	**UCP3**^**−/−**^	**F-value**	**p-value**
Liver	0.42 ± 0.12	0.39 ± 0.18	0.46 ± 0.15	0.38 ± 0.12	0.48	0.69
Muscle	0.78 ± 0.28	0.79 ± 0.24	0.76 ± 0.27	0.75 ± 0.41	0.03	0.99

## Discussion

This study demonstrates that after permanent coronary artery ligation UCP3 genetic deletion is associated with larger infarct size and remodeling and higher cardiac FDG uptake in remote areas as compared to WT mice. These findings suggest that UCP3 deletion induces a metabolic shift that favored glycolytic metabolism. Moreover, the larger area of necrosis and remodeling in response to ischemia in mice leaking UCP3 confirms the cardioprotective role of this protein.

The physiological function of UCP3 is as yet unknown. It has been hypothesized that UCP3 facilitate high rates of fatty acid oxidation [17]. UCP3 is proposed to export the potentially detrimental fatty acid anions to the intermembrane space and cytosol where they can be re-esterified for subsequent use in other pathways. This hypothesis was supported by MacLellan et al. [3] who observed an increased fatty acid oxidation due to augmented UCP3 expression. These results are consistent with the clinical findings of Argyropoulos et al. [18] who demonstrated decreased fat oxidation by indirect calorimetry in a population of Gullah-speaking African Americans with an exon six-splice donor single nucleotide polymorphism in the UCP3 gene. Decreased fat oxidation has also been documented through indirect calorimetry in UCP3^−/−^ mice [19]. These findings support and extend the latter and provide a potential mechanism for the detrimental effects of decreased UCP3 expression in muscle with regard to the development of lipotoxicity and insulin resistance in muscle. Seifert et al. [2] also indicated a role for UCP3 in the adaptation of fatty acid oxidation capacity to fasting and possibly more broadly to perturbed energy balance. In addition, Essop et al. [9] demonstrated a decrease of UCP3 gene expression in rat heart during hypoxia, associated with reduced fatty acid oxidation and increased reliance on glucose metabolism. These data support an overall reduction in the dependence on mitochondrial oxidative phosphorylation in the left ventricle for ATP production in response to hypobaric hypoxia. However, more recent studies have shown that UCP3 is robustly upregulated in skeletal muscle in response to hypoxia [20]. Therefore, the effect of hypoxia on UCP3 expression is still unclear.

UCP3 is expressed in response to reperfusion after ischemia and, activating a mechanism cytoprotective antioxidant, it is capable of reducing the production of ROS and subsequent reperfusion injury [21,22]. In rats it has been shown that the expression of UCP3 is inversely associated with infarct size, probably by activating a protective mechanism to prevent the death of cardiomyocytes in the tissue surrounding the infarcted area [23]. An increased UCP3 expression after ischemia-reperfusion has been demonstrated also in the isolated mouse heart [24] and in the mouse heart in vivo [25]. Therefore, this protein might be a potential therapeutic target for the management of cardiac ischemic disease. During myocardial ischemia, impairment of the energetic activity of the heart is associated with increased level of circulating free fatty acids [26]. However, it has been demonstrated that muscle mitochondrial fatty acid oxidation is decreased in UCP3^−/−^ as compared to WT mice [27]. Thus, it is conceivable that also in the heart the reduced UCP3 levels may lead to a reduction in capacity to oxidize lipids and to in increased glucose consumption. Our data indicate that this metabolic shift is present in remote myocardium where SUV was significantly higher in UCP3^−/−^ than in WT mice (Table 3), indicating the presence of signaling mechanisms between ischemic/necrotic and control remote tissue.

In this study we found that after permanent coronary artery ligation, infarct size and LV volume were significantly greater in UCP3^−/−^ group compared to WT mice. Infarct size is one of the major determinants of post-ischemic cardiac remodeling and adverse outcome. To evaluate cardiac function in WT and UCP3^−/−^ mice after myocardial infarction, transthoracic echocardiography was performed in all experimental groups. Sham-operated UCP3^−/−^ mice showed LV fractional shortening comparable to WT. After myocardial infarction, LV fractional shortening was significantly lower compared to sham-operated mice in both WT and UCP3^−/−^ mice. Moreover, UCP3^−/−^ mice displayed a significant worsening in cardiac function after coronary artery ligation compared with WT.

Mailloux et al. [28] demonstrated that deficiency in UCP3 resulted in a metabolic shift in skeletal muscles that favored glycolytic metabolism, increased glucose uptake and increased sensitivity to oxidative challenge and these findings were confirmed by FDG uptake at PET imaging. To explore whether this metabolic shift towards glycolysis is present also in cardiac muscle, we measured glucose uptake by monitoring myocardial FDG activity in UCP3^−/−^ and in WT mice with and without coronary artery ligation. Our results show no differences in sham-operated animals between WT and UCP3^−/−^. On the other hand, after myocardial infarction SUV in remote areas was higher in both WT and UCP3^−/−^ compared to sham animals. Noteworthy, UCP3^−/−^ mice showed the highest value of SUV and the results of two-way analysis of variance demonstrated a significant interaction between genotype and myocardial infarction. Finally, we found a significant relationship between LV volume and SUV. This finding indicates that adverse remodeling and metabolic derangement are direct related and that UCP3 deletion has an unfavorable impact on both parameters.

This study has some limitations. First, serum glucose levels were not available at time of imaging. In addition, no dynamic acquisition was performed and cardiac glucose metabolism was indexed by SUV. This approach might have been hampered by the systemic effect of UCP3 deletion on glycolytic flux in the whole body tissues. However, in non-cardiac tissue SUV was independent from UCP deletion and myocardial infarction (Table 4), indicating that blood tracer availability for myocardial uptake was not altered in UCP3^−/−^ mice.

## Conclusions

In this study we demonstrate for the first time that, in a mice model of permanent coronary occlusion, UCP3 deficiency results in a metabolic shift that favored glycolytic metabolism and increased FDG uptake in remote areas. We also found a negative remodeling of the left ventricle in response to ischemia in mice leaking UCP3, confirming the cardioprotective role of this protein.

## Abbreviations

UCP: Uncoupling proteins; ROS: Reactive oxygen species; LV: Left ventricular; FDG: Fluorodeoxyglucose; PET: Positron emission tomography; CT: Computed tomography; WT: Wild-type; SUV: Standardized uptake value.

## Competing interests

The authors declare that they have no competing interests.

## Authors’ contributions

SG and MPP performed the statistical analysis and drafted the manuscript. GGS made substantial contribution with statistical analysis. GE, MPe, AB and AC contributed with the conception and design of the study. AG, MPa, ML and MG analyzed the collected data. GE, MPe, AB and AC participated in the study design and interpretation and revised the manuscript critically for important intellectual content. All authors read and approved the final manuscript.

## Pre-publication history

The pre-publication history for this paper can be accessed here:

http://www.biomedcentral.com/1471-2261/14/98/prepub
